# Prediction of Overall Energy Consumption of Data Centers in Different Locations

**DOI:** 10.3390/s22103704

**Published:** 2022-05-12

**Authors:** Yiliu Zhang, Jie Liu

**Affiliations:** 1 School of Computer Science and Technology, Harbin Institute of Technology, Harbin 150001, China; ylzhang@stu.hit.edu.cn; 2 AI Research Institute, Harbin Institute of Technology (Shenzhen), Shenzhen 518055, China

**Keywords:** data centers, energy consumption modeling, power usage effectiveness (PUE), prediction under uncertainty, carbon usage effectiveness (CUE)

## Abstract

The use of big data leads to higher demands for hyperscale data centers (HDCs) in terms of the scale and quantity required for data storage and processing. Before the construction of an HDC, it is necessary to comprehensively analyze the economic budget according to the energy requirements and potential energy cost. We propose a global energy consumption prediction framework based on the power usage effectiveness (PUE) calculation that considers all heat sources and power consumption. The framework integrates physical models and a statistical framework that combines IT equipment energy consumption and data center energy consuming predictions. Furthermore, the framework provides a method to calculate the carbon emissions and electricity cost of the data center. Using hourly meteorological data as climate parameters, combined with a limited range of energy parameters, the annual PUE values of 60 regions were estimated, and a further analysis of the Carbon Usage Effectiveness (CUE) and electricity costs in China was conducted as an example. Based on experimental validation and an evaluation of real-time data, our framework can predict the overall energy consumption of HDCs effectively, filling a gap in HDC research in the Asia-Pacific region and providing a basis for HDC feasibility analysis.

## 1. Introduction

The rapid development of information technology has made data centers key infrastructure to support the development of cloud computing, the Internet of Things, 5 G, AR/VR, etc.; thus, they require significant energy consumption. According to statistics, the global energy demand for data centers surged from 194 TWh to 205 TWh between 2010 and 2018 [1]. The growing demand for computing-intensive services, such as artificial intelligence (AI), and the increasing number of internet users has resulted in exponential growth in both the types and volume of data. Data centers indirectly affect CO${}_{2}$ emissions. It is estimated that, by 2030, CO${}_{2}$ emissions will reach 720 million tons [2]. More HDCs have been constructed to meet this demand, which has caused a contradiction between huge energy consumption and the limited power supply, and it is necessary to balance the performance of HDCs with the capability of the external environment. In addition, operators of HDCs try to find business opportunities by taking advantage of natural advantages [3]. At present, there are two main development directions for large-scale data centers: (1) reducing PUE and improving the energy efficiency of data centers, and (2) purchasing renewable energy to provide power, rather than using a traditional power supply [1]. Therefore, to better optimize data center energy consumption on the premise of application performance, it is necessary to build an appropriate and accurate energy consumption model [4] that incorporates the performance, scale, location, and other characteristics of data centers. Energy consumption models help to predict the consequences of operational decisions, allowing for more effective management and control of a system [5]. We planned to analyze energy consumption by investigating the following two aspects: First, we identified the essential factors affecting energy consumption. Data centers consist of a wide range of complex IT equipment and infrastructures [6], enhancing the complexity of energy consumption calculations. To simplify the calculation, we planned to identify the decisive factors that play key roles in the energy efficiency of a data center to balance the calculation accuracy and efficiency. Second, we built mathematical models for the energy consumption of data centers. Currently, there are no official statistical data on the energy use of HDCs at the global level. It is necessary to formally describe the energy consumption of data centers through mathematical models by transforming the energy efficiency optimization problem of data centers into a classic combinatorial optimization problem [7] to provide optimization strategies.

Data centers of different sizes consume different amounts of energy in the same period. It is meaningless to judge whether a data center meets the energy-saving standard from the perspective of how much energy is consumed. However, PUE can be applied to most scenarios internationally to reflect the energy efficiency of data centers. Traditional data center energy consumption models usually consider all internal components separately and then perform a linear combination. However, it is easy to ignore the interactions between components that are hard to predict. Currently, PUE can be predicted in several ways. Using the PUE formula, we can calculate the overall energy consumption of a data center by measuring the energy consumption of IT equipment.

In this paper, based on the PUE calculation method, we proposed a global energy consumption prediction framework that integrates physical models and a statistical framework and takes all heat generation sources and power consumption components of a data center into consideration. The framework uses the quasi-Monte Carlo (QMC) method [8] to calculate the Sobol sensitivity results [9]. Specifically, the PUE model takes into account the free cooling technology used [10]. Based on hourly meteorological observation data in 60 regions, we estimated the annual PUE of HDCs under the use of different cooling methods and conducted a comparative analysis. We simulated the internal and external parameters of HDCs and predicted the overall energy consumption and carbon emissions of the data center based on PUE and CUE formulas. The feasibility of the zero-carbon data center was then concluded from an in-depth analysis combined with data on the electricity cost and energy structure.

The rest of the paper is organized as follows: Section 2 provides background knowledge and work related to HDC energy consumption technologies. Section 3 presents our energy consumption prediction framework, which includes the PUE models, the IT equipment model, and related calculation methods. Section 4 presents an analysis of the experimental results, an evaluation of our framework, and a discussion. Finally, Section 5 concludes the paper.

## 2. Related Work

Energy-saving, cost reduction, and carbon emission reduction strategies are the current research hotspots regarding the construction of green data centers. The deployment of energy-saving infrastructure and scheduling could dramatically increase the energy efficiency of data centers; however, such infrastructure is required to predict energy consumption. Energy consumption prediction is the basis of data center energy scheduling management, and it can be divided into two parts: the energy consumption of IT facilities and the supporting infrastructure. The former mainly refers to energy consumption during the operation of IT hardware, such as servers, switches, and disk arrays, and the latter mainly includes energy consumption by cooling equipment and power supplies. Generally, the former accounts for a larger proportion of energy than the latter [11,12].

Energy consumption by the data center can be calculated separately. From the perspective of server equipment, the most popular models are the additive model [13,14,15,16] and the model based on system utilization [17,18], and each model type can be further divided into linear and non-linear models. For server energy-saving technologies, the most common technologies include server sleep scheduling [19] and Dynamic Voltage Frequency Scaling (DVFS) [20]. Refrigeration is also a major energy-consuming part of large data centers, as about 40% of the total energy consumption of data centers is used for cooling [21]. For data center refrigeration, heating, ventilation, air conditioning (HVAC), and computer room air conditioning (CRAC) are commonly applied, and many experiments and models have been developed for the accurate estimation of these processes. Some researchers have found that the overall consideration of the IT system layer and the use of a supporting facility layer for cross-layer optimization can maximize the energy-saving potential of the data center [22,23]. This research focused on the cross-layer energy consumption optimization of the data center, taking the modules as a whole and considering the relationships between modules and carrying out unified optimal scheduling and management. The power management methods of a data center air-conditioning system based on IT load scheduling mainly include cooling cost optimization, cabinet heat balancing, peak heat load reduction, and free cooling source utilization, in which free cooling is more universal. Power supply system management technologies based on IT load scheduling mainly include limiting power, the use of UPS supplemental power, and power supply equipment scheduling.

Unified cross-layer energy consumption models are mainly constructed by the following two steps:

I. According to the physical energy consumption characteristics of the equipment, specific mathematical formulas are applied to calculate the energy consumption of each system, a process known as cross-layer joint optimization. The total energy efficiency optimization framework proposed by Wan et al. [24] is one of the examples of cross-layer joint optimization. This framework optimizes the energy cost of a cross-layer data center, spanning the chip layer, server layer, and computer room layer. The thermal prediction model ThermoCast [25] is another example of joint optimization. It can integrate data center sensor observations and physical laws and is capable of capturing cyber–physical interactions and undergoing automatic learning using the data.
II. With the help of learning tools, we can predict the temperature or energy consumption of a system based on current/historical information (load, air conditioning parameters, external environmental parameters, etc.), a process known as prediction-based cross-layer joint optimization. However, methods based on CFD simulation software have high computational complexity. There are also methods based on machine learning, such as the self-aware workload forecasting (SAWF) framework (Hsu et al. [26]), while Gao et al. [27] chose to directly predict the PUE.

PUE [28] is the ratio calculated by the total energy consumption of a data center ($p^{DC}$) over the energy consumption of the IT equipment used ($p^{IT}$). It is an index that is used to evaluate the energy efficiency of a data center, and the result is usually greater than 1. The closer the PUE is to 1, the higher the energy efficiency level of the data center is. PUE is expressed by Equation (1):

(1) $$PUE=\frac{p^{DC}}{p^{IT}}$$

Many researchers have chosen to calculate data center energy consumption by measuring PUE. The most comprehensive study applied a thermodynamic model using constant model inputs to estimate PUE and compared the results with a different free cooling method developed by Gozcü et al. [29]. Research on PUE prediction has also been conducted. A 5-layer neural network developed by Gao’s team [27] mentioned above predicted the PUE of a Google center with an average absolute error rate of 0.004. However, this work used 2-year historical data, and training such a neural network model requires a large amount of nonpublic data containing 19 dimensions, which further increases the training difficulty. Another study by Brady et al. [28] performed high-precision PUE calculations on a set of Facebook HDCs in Prineville, Oregon. This was a thermodynamic modeling case study, which was limited to the airside economizer of a single data center and did not include an accuracy evaluation of other data centers using airside economizers or waterside economizers. Although sensitivity analyses were performed in both studies, the were analyses were conducted separately. The relative importance of parameters was not assessed, and the interaction effects of important variables were also ignored.

In addition to PUE, other energy efficiency metrics have been applied to the evaluation of data centers, such as pPUE [30] (i.e., local PUE) and RER [31] (i.e., renewable energy ratio). Greenpeace, an international environmental protection organization, believes that “green IT = energy efficiency + renewable energy“, which means the greening of the Internet not only needs to involve the reduction of costs by improving the energy efficiency but also requires the use of renewable energy to fundamentally reduce carbon emissions. Internet companies have begun to deploy data centers in places with lower electricity costs or have switched to high usage of new energy, where the power either comes from purchasing from third-party power plants or is provided by new energy power plants built by the company itself. Typical examples of new-energy-powered plants include the solar farm used by Facebook in Ohio for powering data centers and the Green House Data wind farm built in Ohio.

The mature energy consumption prediction methods mentioned above (as well as the currently used method) first establish an energy consumption prediction model based on the historical data generated during the operation of IT equipment and supporting infrastructure and then apply the algorithm to obtain the optimal parameters to control the energy consumption value of various pieces of equipment in the future. The feasibility of these methods relies on the quality of the model. Once the model deviates from the real situation of the equipment operating parameters, the quality of the control strategy cannot be guaranteed.

Cloud computing services are extremely popular and widely adopted due to their flexibility and on-demand advantages. They are hosted in cloud data centers (CDCs), enabling lower energy consumption and carbon emissions. CDC techniques are dependent on geodispersed Modular Data Center (MDC) designs and virtualization-based workload migration [32]. Ahmad et al. [33] reviewed the Virtual Machine (VM)-based workload consolidation schemes in CDCs. P. Nehra et al. [34] compared several existing energy consumption models of CDCs. Yamini et al. [35] proposed a method to reduce the number of servers based on the clique star cover number theorem in which more nodes are connected to the server. Zhang et al. [36] elaborated on the energy consumption in the cloud environment by measuring energy usage in different scenarios. The field of energy efficiency in CDCs holds great promise and remains explorable for researchers.

## 3. Energy Consumption Prediction Framework

To predict the total energy consumption of a data center, we (1) calculated the PUE according to different internal and external parameters, and (2) estimated the IT equipment energy consumption. The overall energy consumption, carbon emissions, and electricity cost of the data center were obtained directly.

### 3.1. PUE Prediction

**Figure 1:**
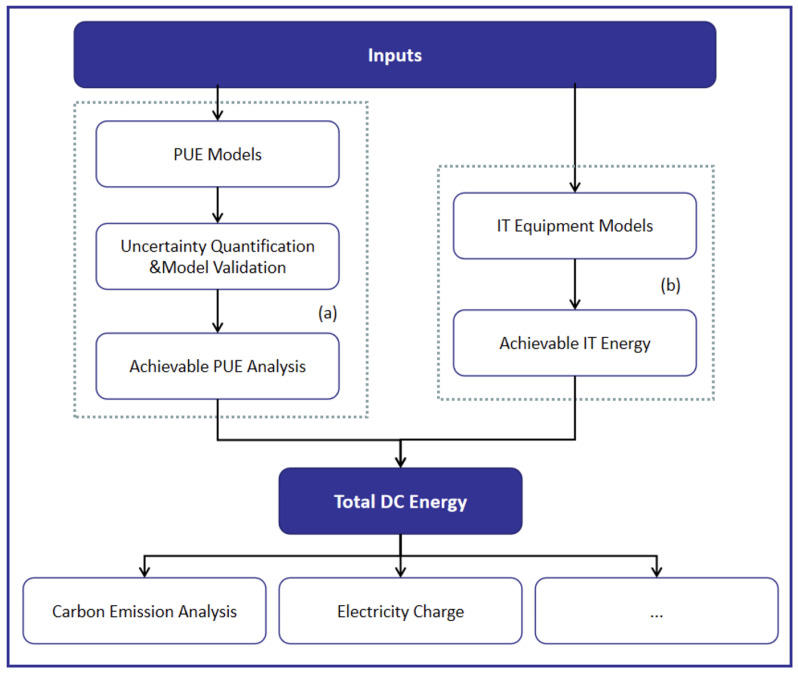
Data center global energy consumption prediction framework. (**a**) is used for PUE prediction, and (**b**) is applied for IT equipment energy consumption.

As shown in Figure 1a, the prediction of PUE is based on the model proposed by Lei et al. [37], which considers all of the heat-generating sources and power-consuming components in the data center system. When free cooling is involved in energy-saving problems, the model mainly considers three cooling scenarios: airside economizers combined with adiabatic cooling (AE), waterside economizers utilizing the evaporative cooling capability of cooling towers (WEC), and waterside economizers using seawater for cooling (WES). When the above technologies cannot provide a sufficient cooling capacity, the mechanical chiller of the cooling system will be deployed to maintain the indoor temperature within an acceptable range.

The aim of the experiments described in this paper was to verify the WEC refrigeration method. Given the external climatic conditions and a specified indoor thermal environment, the PUE model identifies the economizer application scenarios and the amount of additional mechanical cooling that may be required. Based on the thermodynamic model, the total calorific value and electricity consumption of a data center can be described by Equations (2) and (3):

(2) $$q^{DC}=p^{IT}+\left(1-\eta^{UPS}\right)p^{UPS}+\alpha^{PD}p^{PD}+p^{L}$$

(3) $$p^{DC}=p^{IT}+\left(1-\eta^{UPS}\right)p^{UPS}+\alpha^{PD}p^{PD}+p^{L}+\sum_{f}p_{f}^{FAN}+\sum_{p}p_{p}^{PUMP}+p^{CH}$$

where $q^{DC}$ is the total heat generated by a data center. $p^{IT}$, $p^{UPS}$, $p^{L}$, and $p^{DC}$ represent the power used by the IT equipment, UPS, lighting system, and the entire data center, respectively. $p^{PD}$ represents the power used across the power transformation and distribution system. $p_{f}^{FAN}$, $p_{p}^{PUMP}$ represents the power used by the fan type *f* (including CRAC fans and cooling tower fans) and the power used by pump type *p* (including chiller pumps, waterside economizer pumps, cooling tower pumps, and humidification pumps). $p^{CH}$ is the power used by the chiller. The units of all of the above parameters are kW. $\eta^{UPS}$ is the efficiency of the UPS, and $\alpha^{PD}$ is the percentage of power loss in the power transformation and distribution system (i.e., the loss of lines and switches).

The determination of the WEC application scenario requires the water temperature delivered by the economizer heat exchanger ($T_{WEC}$, ${}^{\circ }$C) and the return temperature of the facility water ($T_{rw}$, ${}^{\circ }$C) to be compared. The supply temperature of the facility water ($T_{sw}$, ${}^{\circ }$C) was set according to the dynamically changing temperature of the supply and return air of CRAC, which is described by Equation (4):

(4) $$T_{sw}=T_{ra}-\left(T_{ra}-T_{sa}\right)/\epsilon =T_{ra}-\Delta T_{air}/\epsilon$$

where $T_{ra}$ represents the CRAC supply air temperature, and $T_{sa}$ represents the CRAC supply air temperature. $\Delta T_{air}$ is the temperature difference between the supplied and returned CRAC air. The units of the above parameters are ${}^{\circ }$C. ϵ is the heat exchanger effectiveness of CRAC cooling coils. Then, $T_{rw}$ can be calculated as the temperature difference between the supplied and returned facility water ($\Delta T_{w}$, ${}^{\circ }$C). $T_{WEC}$ can be calculated by Equation (5):

(5) $$T_{WEC}=T_{wb}+\left(AT_{CT}-AT_{EX}\right)/\epsilon =T_{ra}-\Delta T_{air}/\epsilon$$

where $T_{wb}$, $AT_{CT}$, and $AT_{EX}$ represent the wet bulb temperature of outdoor air, the approach temperature of the cooling tower, and the approach temperature of the economizer heat exchanger, respectively. Their units are ${}^{\circ }$C.

In general, $q^{DC}$ can be expressed by Equation (6):

(6) $$q^{DC}=q^{WEC}+q^{CH}$$

where $q^{WEC}$ represents free cooling supplied by WEC. Its units are kW.

**Figure 2:**
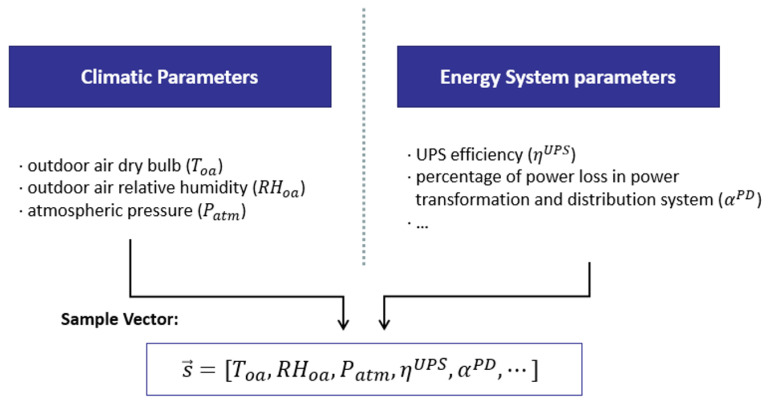
Generation of example vectors.

As shown in Figure 2, the example vectors of the PUE model can be expressed in vector form:

(7) $$\vec{s}=\left[T_{oa},RH_{oa},P_{atm},\eta^{UPS},\alpha^{PD},\ldots \right]$$

The input of the PUE model can be divided into two categories: climate parameters and data center energy system parameters. The latter includes equipment specifications, system operating efficiency parameters, and indoor environmental set-points, and a detailed description of the former can be found in [37].

In order to find the parameters that have the greatest influence on predicting the PUE value and to further evaluate the influence of the interactions among variables during PUE prediction, we first used Sobol’s method to generate sample vectors from climate and energy system parameters, processed the sample vectors using the model, and finally, calculated the Sobol sensitivity index using the quasi-Monte Carlo (QMC) method for the uncertainty analysis. If the uncertainty of key parameters can be reduced, the accuracy of the prediction results can be greatly improved.

The outdoor dry bulb temperature and outdoor relative humidity ranges were set to −40–40 ${}^{\circ }$C (approximate range of the outdoor dry bulb temperature in all regions of China throughout the year) and 0–100%. The range of parameters was estimated and determined based on public information, and each interval adopted a uniform probability distribution.

The results of the Sobol sensitivity analysis show that climate parameters play a key role in PUE values, so site-specific climate data are needed as input. Fortunately, accurate values are relatively easy to obtain with meteorological data as the input. Specific data on energy and machinery depend on the internal documents of the specific data center. The results of the analysis of the Sobol sensitivity are described in detail in Section 4.1.

### 3.2. IT Equipment Energy Consumption Model

Energy consumption models of IT equipment presented in previous work can be roughly divided into two categories: energy consumption evaluation models based on system utilization and energy consumption prediction models based on performance monitoring counters (PMC) [38]. We built an additive model for IT equipment energy consumption considering the former category, as described by Equation (8):

(8) $$p^{IT}=p^{CPU}+p^{MEM}+p^{DISK}+p^{Others}$$

Specifically, processor energy consumption can be calculated by CPU usage modeling, memory energy consumption can be calculated by cache miss rate modeling, and disk energy consumption can be calculated as the number of read and write bytes. Based on the performance monitoring counter, the model was built as described by Equation (9):

(9) $$p^{IT}=C_{0}+\sum_{i=1}^{n}C_{i}E_{i}$$

where $C_{0}$ is a constant, $E_{i}$ is the collected performance counter event, and $C_{i}$ is the influence coefficient of the $i^{th}$ event on energy consumption. $C_{0}$ and $C_{i}$ can be found by linear regression.

Energy prediction models based on PMC have become mainstream applications for energy optimization. They always outperform energy modeling methods based on system utilization due to their fine-grained characteristic. A. Shahid et al. pointed out that any nonlinear energy model using only PMC (such as RF and NN models) is inconsistent and inaccurate [39] and proposed a theoretical framework for computing energy prediction models [40] because of the current state-of-the-art multicore CPU energy prediction models based on linear regression.The basic practical implications of the theory include selection criteria for model variables, model intercepts, and model coefficients. The model theory follows the physical laws of the conservation of computing energy.

Property 1: An abstract application run can be accurately characterized by a set of n-vectors of PMCs over R≥0. A null vector of PMCs is represented by

(10) $$\mathrm{NULL}={\left\{0\right\}}_{k=1}^{n}$$

A function, $f_{E}:R_{\geq 0}^{n}\overset{}{\to }R_{\geq 0}$ maps the vectors to energy values, and $\forall p,q\in R_{n}\geq 0$,

(11) $$p=q\Rightarrow f_{E}\left(p\right)=f_{E}\left(q\right)$$

Property 2: There exists an application space, (A,⊕), where A is a (infinite) set of applications, and ⊕ is a binary function on A,⊕:

(12) $$A\times A\overset{}{\to }A$$

There exists a (infinite) set of binary operators,

(13) $$O=\{\circ_{PQ,k}:R_{\geq 0}\times R_{\geq 0}\overset{}{\to }R_{\geq 0},P,Q\in A,k\in \left[1,n\right]\}$$

so that for each P,Q∈A, and their PMC vectors $p={\left\{p_{k}\right\}}_{k=1}^{n},q={\left\{q_{k}\right\}}_{k=1}^{n}\in R_{\geq 0}^{n}$, respectively, the PMC vector of the compound application P⊕Q will be equal to ${\left\{p_{k}\circ_{PQ,k}qk\right\}}_{k=1}^{n}$.

Property 3:

(14) $$f_{E}\left(\mathrm{NULL}\right)=0$$

Property 4:

(15) $$\forall p\in R_{\geq 0}^{n}\wedge \neq \mathrm{NULL},f_{E}\left(p\right)>0$$

Property 5: $\forall P,Q\in A,p={\left\{p_{k}\right\}}_{k=1}^{n},q={\left\{q_{k}\right\}}_{k=1}^{n}\in R_{\geq 0}^{n},\circ_{PQ,k}\in O,$

(16) $$f_{E}\left({\left\{p_{k}\circ_{PQ,k}q_{k}\right\}}_{k=1}^{n}\right)=f_{E}\left(p\right)+f_{E}\left(q\right)$$

When $f_{E}\left(x\right)$ is a linear function, the model is linear. The linear consistent energy prediction model can be formalized as $\forall p={\left(p_{k}\right)}_{k=1}^{n},p_{k}\in R_{\geq 0},$

(17) $$f_{E}\left(p\right)=\beta_{0}+\beta \times p=\beta_{0}+\sum_{k=1}^{n}\beta_{k}\times p_{k}$$

where $\beta_{0}$ is the model intercept, $\beta =\{\beta_{1},\beta_{2},\ldots ,\beta_{n}\}$ is the vector of the regression coefficients or the model parameters. Influenced by measurement errors or stochastic noise, the measured energy can be described by Equation (18):

(18) $${\tilde{f}}_{E}\left(p\right)=f_{E}\left(p\right)+\epsilon$$

where the error term ϵ is a Gaussian random variable with an expectation of zero and variance of $\sigma^{2}$, written as $\epsilon \;N(0,\sigma^{2})$.

Linear energy models have following properties:

**Theorem** **1.** *If a linear energy predictive model, such as Equation (17), is consistent, the model intercept must be zero and the model coefficients must be positive.*

**Theorem** **2.** *If a consistent energy model is linear, then it is strongly composable with O={+}.*

**Theorem** **3.** *If a consistent energy model is strongly composable with O={+} and the function $f_{E}\left(x\right)$ is continuous, then it is linear.*

Details and proofs can be found in [40]. Experiments on two modern Intel multicore servers improved the prediction accuracy of state-of-the-art linear regression models with significant energy saving. This theory can be used to build accurate linear energy prediction models.

Based on the above settings, it can be assumed that the PMC-based energy prediction model satisfying the following five properties of the extended model can be defined as a consistent energy model under the same computing environment.

### 3.3. Calculation of the Total Energy Consumption and Related Analysis

According to Equation (1), we can calculate the total energy consumption of a data center using Equation (19):

(19) $$p^{DC}=PUE\times p^{IT}$$

If the PUE result is predicted by Section 3.1, and the IT equipment energy consumption is obtained by Section 3.2 or known data, the total energy consumption of a particular data center can be inferred.

PUE cannot evaluate the environmental performance and energy expenditure of a data center. The Green Grid Organization proposed a new energy measurement standard for green data centers, the Carbon Usage Effectiveness (CUE). The CUE is the carbon emission intensity per kilowatt-hour of electricity used [41], the ratio of the total CO${}_{2}$ emissions of the data center ($D_{total}$, kgCO${}_{2}$eq) to the energy consumption of the IT equipment ($p^{IT}$, kW × h):

(20) $$CUE=\frac{D_{total}}{p^{IT}}$$

The CUE can also be expressed by the product of the Carbon Emission Factor (CEF) and PUE as shown in Equation (21):

(21) CUE=CEF×PUE

The CEF is the carbon emissions per unit of energy consumed (kgCO${}_{2}$e × kWh${}^{-1}$), and Table 1 shows the CEF of several common electrical energy sources [42]. The carbon emission factor of fossil fuels is the largest.

Of the energy sources presented above, wind energy and solar energy are the most promising green energy sources for data centers due to their extensive existence and environmental friendliness. However, the power generation of these green energy sources varies over time, causing instability. Environmental conditions also have a great impact. For example, wind speed affects wind energy, and sunshine intensity affects solar energy. In terms of early installation and deployment, new energy power generation costs more than energy production by traditional fossil fuel power plants, but the former has lower follow-up management costs and significantly less pollutant emissions during operation.

Table 2 shows the CEF and overheads of the grid and some new green energy products. In terms of the electricity cost, it is necessary to consider the power source. In addition, the impact of its carbon emissions needs to be considered.

## 4. Evaluation

We used hourly meteorological data as the input data for the climate component of the PUE model and generated random values within the range of established reliable mechanical parameters as the parameter input for the energy component. The annual PUE was estimated, and the carbon emissions and electricity costs were further analyzed.

### 4.1. Sensitivity Results

**Figure 3:**
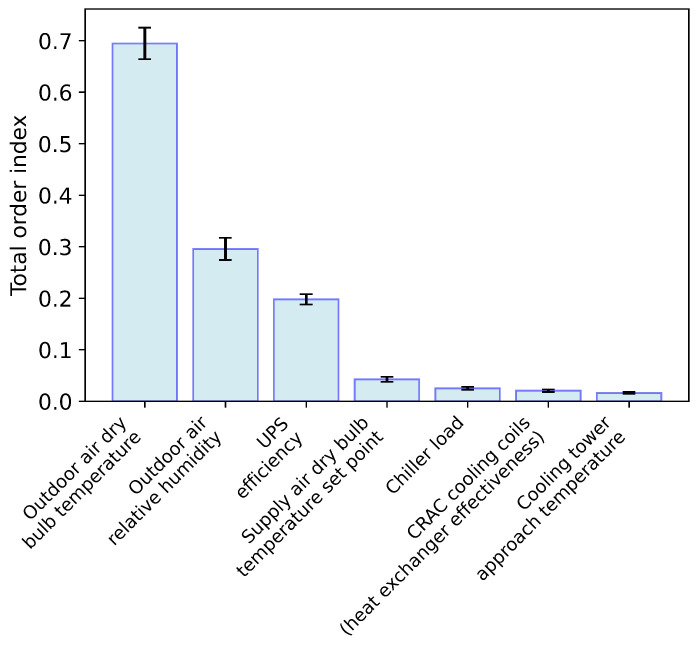
Total order sensitivity indices greater than 0.01.

Making the key input parameters as accurate as possible is an important way to reduce the uncertainty of model prediction. As the largest source of uncertainty, climate parameters can be obtained from weather databases in most parts of the world. These climate data are exact sensor data and are beneficial to the model’s accuracy. However, the internal parameters of data centers are hard to obtain, and the specific internal settings of the data center, such as the UPS efficiency, may be difficult to determine. The accuracy of the method was assessed by the bootstrapping method using 100 sample replacements to calculate the 95% confidence interval of the sensitivity indicator [43]. Results of all sensitivity indices are shown in the attached Table A1, and factors greater than 0.01 are shown in the following Figure 3:

To show the interaction effect of the variables, we divided the sum of the total order sensitivity indices ($\sum_{i=1}^{k}S_{T_{i}}$) by the sum of the first order sensitivity indices ($\sum_{i=1}^{k}S_{i}$). The ratio was 1.96, which proves that the total order sensitivity should be used, because the global sensitivity analysis takes the interactions between parameters into account, making it more robust than the local sensitivity analysis, while the first order sensitivity index can only reflect the effect of a single variable [44].

This section discusses the sensitivity analysis results obtained under Chinese climatic conditions (Section 3.1). When applying the model to other regions, a sensitivity analysis based on the climatic characteristics unique to that region would need to be performed.

### 4.2. Annual PUE Estimation Analysis

**Figure 4:**
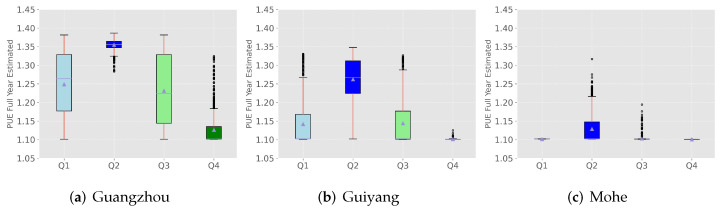
Annual PUE estimation in 3 cities.

Based on the annual PUE values obtained from hourly meteorological data, we drew boxplots by season. Figure 4 lists the results for Guangzhou, Guiyang, and Mohe.

**Figure 5:**
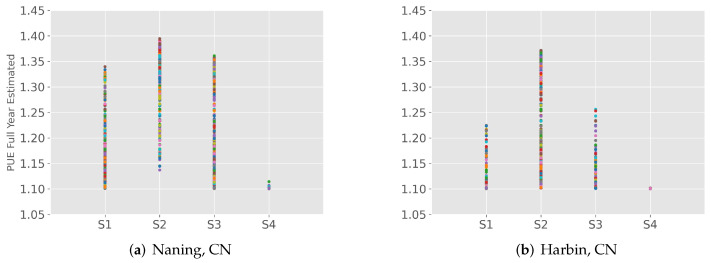
Typical annual PUE estimation of 2 cities in China by season.

Figure 4 shows that the average PUE value is smaller in places where the average annual temperature is lower. The uncertainty range of the annual simulation results is large. Therefore, it is more intuitive to distinguish by season: a higher PUE in summer (S2) and a lower PUE in winter (S4). Figure 5 shows scatter plots of the estimated PUE values for Nanjing and Harbin by quarter. Overall, Nanjing’s estimated PUE is higher than Harbin’s. Obviously, the temperature is higher in summer, and the PUE values of the two cities in summer are relatively high, while those in the first quarter are lower. In the fourth quarter, the difference in PUE estimates between the two cities is even more pronounced, indicating that the large-scale air-cooled data center built in Harbin has better cooling conditions while utilizing free cooling sources. This can also be applied to other regions, which means that in regions with relatively higher temperatures and humidity levels, the energy consumption required by data centers is greater. In fact, reports of PUE values measured in existing data centers confirm this issue. Lei et al. [37] compared and evaluated models using 17 HDCs data from Google and Facebook. Most of the model prediction results controlled the prediction interval within 50%, and almost all values were within the 90% prediction interval, ensuring the accuracy of the PUE prediction model. Chinese data centers usually adopt a cooling method that combines a cooling tower and a plate heat exchanger. However, since real-time or hourly tracking PUE data from Chinese data centers has not been released, the prediction results of the annual PUE (WEC) value in this paper were compared with actual data reported from different places to confirm the accuracy of this model.

**Figure 6:**
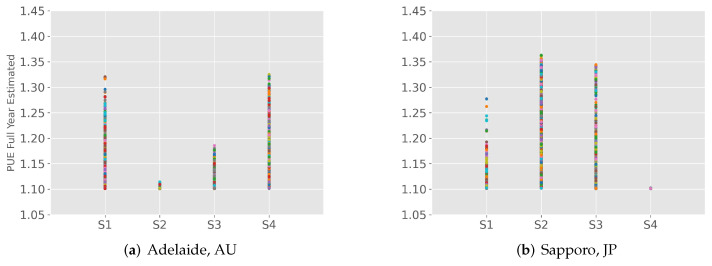
Typical annual PUE estimation of 2 cities worldwide by season.

The model has been verified with data from several countries, but no one has applied it to the Asia-Pacific region. We predicted annual PUE values for some cities in Australia, Japan, and Russia. Taking Adelaide and Sapporo as examples in Figure 6, we modified the parameters according to the actual local conditions, and the results are in line with the reported values and our expectations.

We also conducted experiments on some regions in the US not mentioned in [37]. All scatter plots are shown in Appendix A.

### 4.3. Carbon Emissions Prediction

This subsection and Section 4.4 discuss China as an example. The analysis method for other regions is the same.

Table 3 was taken from the “Research Report on China’s Carbon Neutrality Before 2060“ released by the Global Energy Interconnection Development Cooperation Organization (GEIDCO) [45]. Assuming that the proportion of energy used by the data center is similar to the data in the table, the carbon emissions can be roughly estimated. If the CEF of biomass and other energy sources is considered to be 10 gCO${}_{2}$eq × kWh${}^{-1}$, and the carbon emissions of oxygen-fired units are considered, then in 2020, 2030, and 2060, the estimated CUE of the data center will be 506.705x, 311.739x, 53.738x PUE respectively.

It can be inferred that the estimated carbon emission of data centers in China in 2030 will be about 60% of that in 2020, and emissions are expected to reduce by nearly 90% CO${}_{2}$ by 2060 without the consideration of climate change and the optimization of energy saving technology in data centers. By substituting the predicted PUE values of different regions, it is also possible to carry out a comparative analysis of different regions.

COVID-19 has highlighted the important roles of digital technology, the digital industry, and digital services in the operation of the economy and society. In the postepidemic era, people’s production and lifestyles have undergone profound changes. The numbers of data centers and racks have increased dramatically, and electricity demand has grown rapidly. Ensuring an increase in clean energy installations and making them generate as much power as possible are essential to decarbonize the entire electricity industry. The spatiotemporal controllability of part of the power load in HDCs is conducive to the promotion of renewable energy consumption. Coal power harms the environment and contributes to climate change, and its economic benefits are not very good. Therefore, coal removal is the most direct and effective measure for greening and the attainment of a low-carbon power structure.

### 4.4. Electricity Cost Estimation

In order to simplify the calculation, we used general industrial and commercial sales prices under 10 kV form various provinces and cities in 2019 as the electricity fee calculation parameters (Table 4). The data were collected from the local Development and Reform Commission, the Price Bureau, and other departments.

Assuming that the total annual energy consumption of IT equipment in the preconstructed data center was 100 million kWh, the calculation was performed using the estimated PUE for each region in 2019. For situations where new HDCs are built in various places, the estimated electricity costs are shown in Table 5. The top 5 regions in descending order are presented here, and the full dataset is presented in Table A2 in Appendix A.

As shown in Table 5, even if some areas consume less energy, the calculated electricity cost is relatively high due to the higher electricity price. The top 5 regions in ascending order are shown here, the full dataset is presented in Table A3.

## 5. Conclusions

This study proposed a framework to predict the overall energy consumption of HDCs with air-cooled IT equipment. According to the PUE predicted from the location and the internal structure of data centers from the point of view of IT equipment energy consumption, the total energy consumption can be calculated, and the carbon emissions and electricity costs can be forecast. Using the hourly meteorological data in the NOAA Integrated Surface Database (ISD) as climate parameters, the annual PUE values and the electricity cost of data centers to be built in 49 regions in China were analyzed. We also conducted an experiment involving 11 regions in other countries to extend the generality of our framework. Compared with the data presented in actual reports, our framework performed well. Our results show that climate is an important factor that impacts the energy consumption of data centers with consideration of free cooling. Generally, building HDCs in areas with lower temperatures takes advantage of free cooling and could save energy costs and improve the economic efficiency. The UPS efficiency also has a large impact on the results of the model. Data centers can improve their overall energy efficiency by increasing the efficiency of the UPS. Compared with [37], we found that when some parameters are modified according to the characteristics of regions, the sensitivity indices and their sequence will change. This reflects the impact of location factors on data center construction.

According to the results, some regions have low annual PUE values with high electricity costs and unreasonable energy structures. This means that PUE should not be seen as the only criterion for measuring the quality of data centers. With the improvement of policies and people’s awareness of environmental protection, the cost of carbon trading and climate change need to be considered in the construction of data centers as well. Therefore, it is necessary to coordinate the cost factors when considering the construction of HDCs and consider the comprehensive benefits, social impact, and environmental friendliness in general. 

## Figures and Tables

**Table 1:** CEF of common electric energy sources.

Energy Type	Carbon Emission Factor / kgCO${}_{2}$e × kWh${}^{-1}$
Coal	968
Oil	890
Natural Gas	440
Solar Energy	53
Wind Energy	29
Nuclear Energy	15
Water	13.5

**Table 2:** Unit energy expenditure and carbon emission factor.

Energy	Price per Unit/$ × kWh${}^{-1}$	Carbon Emission Factor/kgCO${}_{2}$e × kWh${}^{-1}$
Electricity Grid	5.0	586
PPA	6.0	0
REC	0.5	0
DG	30.0	1056

**Table 3:** Installed power generation and structure in China from 2020 to 2060 (in 100 million kWh).

Energy Type	2020		2030		2060	
	Generation	Proportion	Generation	Proportion	Generation	Proportion
Wind	2.8	12.7%	8	21%	25	31.2%
Solar	2.5	11.3%	10.25	27%	38	47.4%
Hydro	3.7	16.8%	5.54	14.6%	7.6	9.5%
Coal	10.8	49%	10.5	27.6%	0	0.0%
Gas	0.98	4.5%	1.85	4.9%	3.2	4.0%
Nuclear	0.5	2.3%	1.08	2.8%	2.5	3.1%
Biomass	0.67	3%	0.82	2.2%	1.8	2.2%
Oxygen	0	0%	0	0%	2	2.5%
Total	22		38		80	

**Table 4:** Current electricity prices for general industrial and commercial use in various regions.

Area	Current Electricity Price ^1^/$ × kWh${}^{-1}$
Jilin	0.1183
Beijing	0.1138
Shanghai	0.1126
Hubei	0.1116
Hunan	0.1106

^1^ "$" here is the price of USD in 2022.

**Table 5:** Top 5 estimated average annual electricity cost of data centers in various regions.

Area	Electricity Cost ^1^/$1M per Year
Delinha	8.1376
Xining	8.1429
Kashgar	8.1903
Karamay	8.2474
Yinchuan	9.6742

^1^ "$" here is the price of USD in 2022.

**Figure A1:**
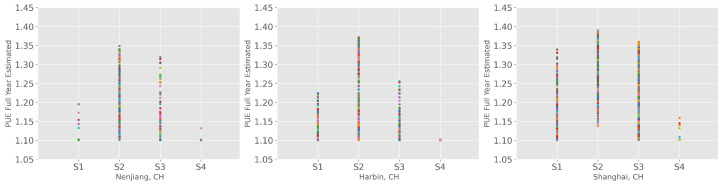

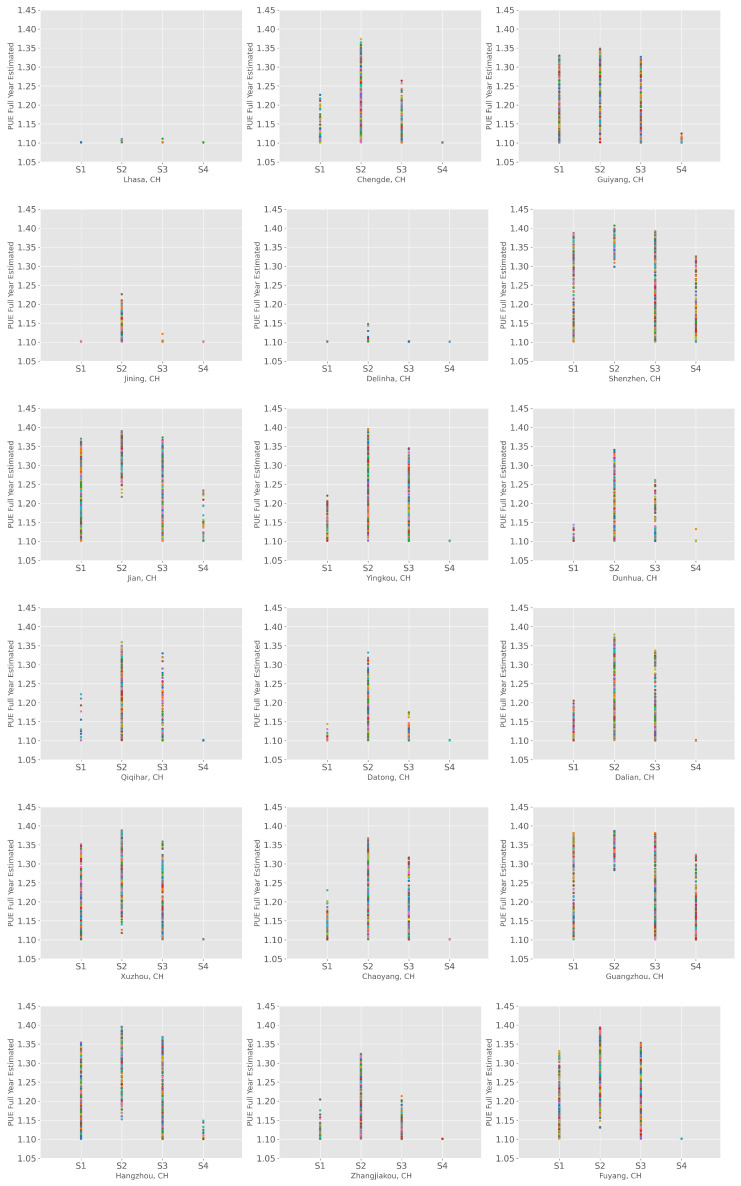

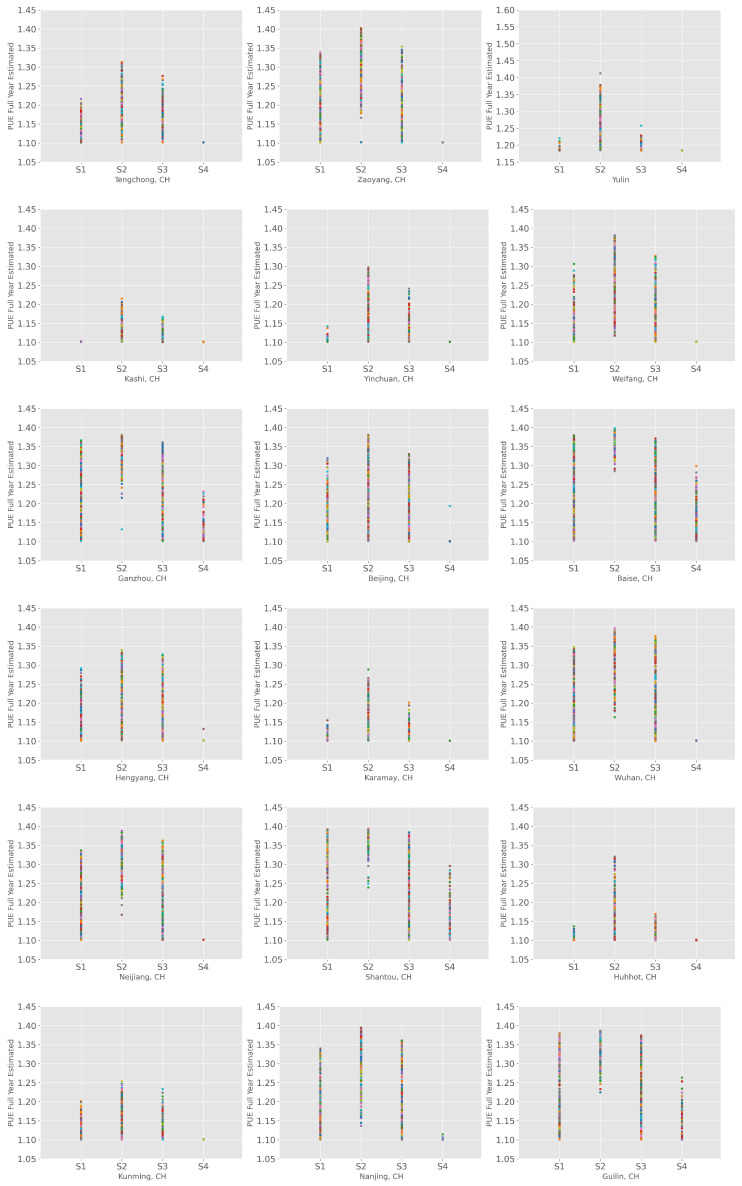

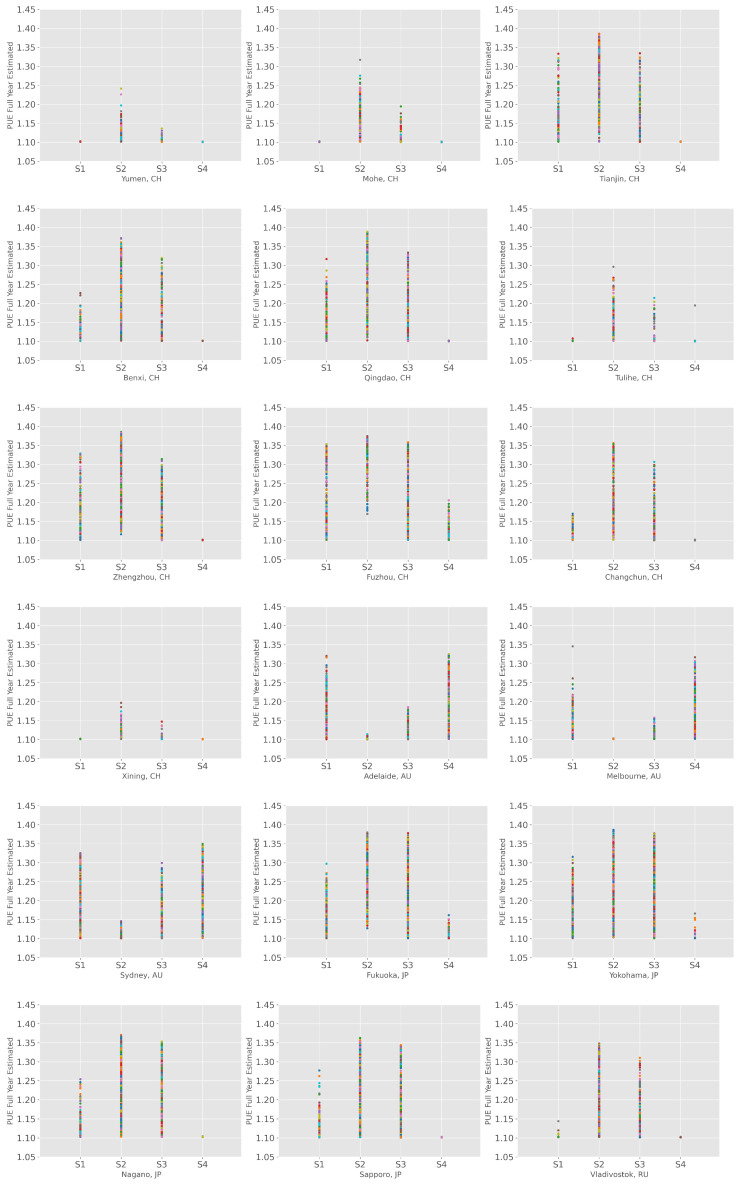

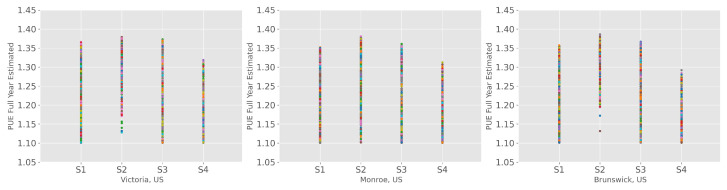
Annual PUE estimation.

## Data Availability

[Integrated Surface Dataset (Global)] NOAA National Centers for Environmental Information. 2001. Integrated Surface Dataset (Global); https://www.ncei.noaa.gov/access/metadata/landing-page/bin/iso?id=gov.noaa.ncdc:C00532; NCEI DSI 3505_03.

## Appendix A

**Table A1:** Sobol Sensitivity Index (WEC).

Total Sensitivity Index	
Input	Index
Outdoor Dry Bulb Temperature	7.17 × 10^−1^
Outdoor Relative Humidity	3.07 × 10^−1^
UPS efficiency	2.02 × 10^−1^
Supply air dry bulb set point	4.38 × 10^−2^
Chiller partial load factor	2.64 × 10^−2^
Heat exchanger effectiveness (CRAC cooling coils)	2.05 × 10^−2^
Approach temperature (cooling tower)	1.53 × 10^−2^
Percentage of power loss in power transformation and distribution system	8.27 × 10^−3^
Temperature difference (supply/return facility system water)	7.04 × 10^−3^
COP relative error to regressed value	3.72 × 10^−3^
Fan pressure (CRAC)	3.60 × 10^−3^
Temperature difference (supply/return CRAC)	3.29 × 10^−3^
Fan efficiency (CRAC)	2.39 × 10^−3^
Temperature difference (supply/return cooling tower water)	1.65 × 10^−3^
Fan pressure (cooling tower)	1.51 × 10^−3^
Liquid–gas ratio (cooling tower)	1.43 × 10^−3^
Atmospheric pressure	1.30 × 10^−3^
Approach temperature (economizer heat exchanger)	1.06 × 10^−3^
Pump pressure (cooling tower)	7.36 × 10^−4^
Pump efficiency (cooling tower)	3.84 × 10^−4^
Sensible heat ratio (SHR)	2.05 × 10^−4^
Pump pressure (waterside economizer pump)	1.42 × 10^−4^
Fan efficiency (cooling tower)	1.31 × 10^−4^
Lighting power to IT power ratio	7.94 × 10^−5^
Pump efficiency (waterside economizer pump)	7.43 × 10^−5^
Pump pressure (chiller pump)	1.42 × 10^−5^
Pump efficiency (chiller pump)	7.12 × 10^−6^
Pump efficiency (humidification pump)	3.09 × 10^−8^
Pump pressure (humidification pump)	1.50 × 10^−8^
Total	1.369
**First Order Sensitivity**	
**Input**	**Index**
Outdoor Dry Bulb Temperature	4.20 × 10^−1^
UPS efficiency	2.02 × 10^−1^
Outdoor Relative Humidity	4.29 × 10^−2^
Supply air dry bulb set point	8.15 × 10^−3^
Percentage of power loss in power transformation and distribution system	8.06 × 10^−3^
Fan pressure (CRAC)	3.56 × 10^−3^
Fan efficiency (CRAC)	2.51 × 10^−3^
Chiller partial load factor	2.38 × 10^−3^
Heat exchanger effectiveness (CRAC cooling coils)	2.23 × 10^−3^
Temperature difference (supply/return facility system water)	2.08 × 10^−3^
Temperature difference (supply/return cooling tower water)	1.77 × 10^−3^
Fan pressure (cooling tower)	1.44 × 10^−3^
Atmospheric pressure	1.08 × 10^−3^
COP relative error to regressed value	9.52 × 10^−4^
Liquid–gas ratio (cooling tower)	7.90 × 10^−4^
Pump efficiency (cooling tower)	3.14 × 10^−4^
Sensible heat ratio (SHR)	2.67 × 10^−4^
Temperature difference (supply/return CRAC)	1.92 × 10^−4^
Lighting power to IT power ratio	1.61 × 10^−4^
Approach temperature (economizer heat exchanger)	1.47 × 10^−4^
Pump pressure (waterside economizer pump)	1.26 × 10^−4^
Pump efficiency (waterside economizer pump)	4.67 × 10^−5^
Pump efficiency (chiller pump)	1.28 × 10^−5^
Pump pressure (chiller pump)	1.14 × 10^−5^
Pump pressure (humidification pump)	4.24 × 10^−8^
Pump efficiency (humidification pump)	−1.68 × 10^−8^
Fan efficiency (cooling tower)	−6.52 × 10^−5^
Approach temperature (cooling tower)	−1.63 × 10^−3^
Total	0.700

**Table A2:** Current electricity price for general industrial and commercial use in various regions.

Area	Current Electricity Price ^1^/$ × kWh${}^{-1}$
Jilin	0.1183
Beijing	0.1138
Shanghai	0.1126
Hubei	0.1116
Hunan	0.1106
Heilongjiang	0.1088
Zhejiang	0.1086
Inner Mongolia (East)	0.1084
Hainan	0.1076
Tianjin	0.1074
Liaoning	0.1070
Guangdong	0.1061
Sichuan	0.1057
Guangxi	0.1049
Jiangsu	0.1045
Chongqing	0.1023
Anhui	0.1020
Jiangxi	0.1007
Shaanxi	0.0985
Gansu	0.0976
Shandong	0.0970
Henan	0.0948
Fujian	0.0937
Guizhou	0.0926
Hebei (South)	0.0844
Inner Mongolia (West)	0.0853
Shanxi	0.0835
Yunnan	0.0835
Hebei (North)	0.0831
Ningxia	0.0805
Xinjiang	0.0688
Qinghai	0.0687

^1^ "$" here is the price of USD in 2022.

**Table A3:** Estimated average annual electricity cost of data centers in various regions.

Area	Electricity Cost ^1^/$1 M per Year
Delinha	8.1380
Xining	8.1433
Kashgar	8.1907
Karamay	8.2478
Yinchuan	9.6746
Datong	10.0073
Zhangjiakou	10.0087
Kunming	10.0599
Chengde	10.1136
Jining	10.1335
Hohhot	10.2079
Tengchong	10.2222
Guiyang	11.5760
Yumen	11.5923
Yulin	11.7599
Zhengzhou	11.8100
Weifang	12.0848
Qingdao	12.0895
Fuzhou	12.1301
Lhasa	12.5513
Turi	12.8846
Fuyang	12.9170
Mohe	12.9731
Gian	13.0884
Nenjiang	13.1002
Chaoyang	13.1083
Ganzhou	13.1305
Benxi	13.1563
Qiqihar	13.1922
Xuzhou	13.1992
Dalian	13.2328
Harbin	13.2465
Nanjing	13.2875
Yingkou	13.2930
Tianjin	13.3773
Neijiang	13.5718
Guilin	13.7005
Hengyang	13.7051
Hangzhou	13.9360
Baise	14.0683
Beijing	14.0985
Zaoyang	14.1248
Guangzhou	14.1670
Dunhua	14.2605
Wuhan	14.2751
Shantou	14.2795
Shanghai	14.3534
Changchun	14.4077
Shenzhen	14.4802

^1^ "$" here is the price of USD in 2022.
